# Physical activity and lung cancer screening (PALS): feasibility randomised controlled trial of exercise and physical activity in lung cancer screening

**DOI:** 10.1186/s12931-025-03158-0

**Published:** 2025-03-06

**Authors:** Asha Bonney, Catherine L. Granger, Daniel Steinfort, Cameron Patrick, Henry M. Marshall, Kwun M. Fong, Renee Manser

**Affiliations:** 1 Department of Medicine, University of Melbourne, Melbourne, VIC Australia; 2 Department of Respiratory and Sleep Medicine, The Royal Melbourne Hospital, 300 Grattan Street Parkville, Melbourne, VIC Australia; 3 Department of Physiotherapy, The Royal Melbourne Hospital, Melbourne, VIC Australia; 4 Department of Physiotherapy, University of Melbourne, Melbourne, VIC Australia; 5 Statistical Consulting Centre, School of Mathematics and Statistics, The University of Melbourne, Melbourne, Australia; 6 Thoracic Research Centre, The University of Queensland, Brisbane, QLD Australia; 7 Department of Thoracic Medicine, The Prince Charles Hospital, Brisbane, QLD Australia

**Keywords:** Exercise, Lung cancer, Prevention, Screening

## Abstract

**Background:**

There is increasing evidence that screening provides a catalyst for behavioural change. Low physical activity (PA) levels are a potentially modifiable risk factor for developing lung cancer. This study aims to assess the feasibility and safety of a semi-supervised 8-week multi-modal exercise program to improve health-related quality of life and PA levels of participants of lung cancer screening.

**Methods:**

Participants without lung cancer from a single Australian International Lung Screen Trial (ILST; NCT02871856) site were invited to this feasibility randomised controlled trial. Enrolled participants were randomised to usual care, written material, or a home-based exercise program (in addition to written material). Assessments occurred at baseline, 9 weeks, and 6 months.

**Results:**

75 participants were enrolled over a 3-month period in 2022 (consent rate of 67%). 43% of participants were female, median age 66 years old (IQR 62, 73). Of the 25 participants randomised to the home-based exercise program, 22 participants (88%) attended > 70% of weekly sessions. 99% (74/75) of study participants attended their 9-week and 6-month follow-up assessments.

**Conclusions:**

This study confirms the feasibility and high compliance of delivering a semi-supervised 8-week multi-modal exercise program to participants of a lung cancer screening program. It was safe, with no adverse events.

**Clinical trial registration:**

Australian Clinical Trials Register https://www.australianclinicaltrials.gov.au ACTRN12622001001785.

**Supplementary Information:**

The online version contains supplementary material available at 10.1186/s12931-025-03158-0.

## Background

Lung cancer is the leading cause of cancer-related death globally [1], and has significant impacts on functional status and health-related quality of life (HRQoL) [2, 3]. Lung cancer screening with low-dose computed tomography (LDCT) has been demonstrated to reduce lung cancer mortality in high-risk (people with active smoking history) populations by detecting lung cancer at an earlier stage which is associated with better prognosis [4]. Consequently, a growing number of countries are implementing LDCT lung cancer screening programs, including Australia, Croatia, Czechia, Poland, South Korea, Taiwan, United Kingdom, and the United States of America [5].

Screening is increasingly being recognised as an opportunity for behaviour change, and as such, smoking cessation support is already strongly recommended as part of lung cancer screening programs and trials [6]. However, another potentially modifiable risk factor for lung cancer is low physical activity (PA) levels [7]. Several systematic reviews have evaluated the relationship between PA and lung cancer [7–9]. They demonstrated an inverse relationship between PA levels and lung cancer risk in people with active smoking histories, with the effect of PA persisted when adjusted for smoking intensity and duration [7]. This could be potentially explained by the biological differences in lung tumorigenesis between people with an active tobacco exposure and people without [10]. Whilst the underlying mechanisms by which low PA levels contribute to lung cancer development are not clearly established, several mechanisms have been proposed by which exercise reduces oxidative damage contributing to carcinogenesis [11].

PA is also associated with better heart health, mood, overall wellbeing, a lower risk of diabetes, reduced smoking cravings and withdrawal symptoms, as well as a lower risk of other cancers [12, 13]. Improvements in heart health are particularly significant as the biggest cause of death in the National Lung Screen Trial, the largest LDCT lung cancer screening randomised controlled trial (RCT) to date, was cardiac disease [14]. Additionally, in people with lung cancer, higher levels of PA are associated with improved exercise capacity, health-related quality of life (HRQoL), mood, and physical function [3, 15].

The World Health Organisation (WHO) recommends adults (aged ≥18 years old) should do at least 150 to 300 min of moderate-intensity aerobic PA or at least 75 to 150 min of vigorous intensity aerobic PA, or an equivalent combination of moderate and vigorous PA intensity throughout the week [12]. At least 2 days per week of muscle-strengthening activities and limiting the amount of sedentary time are also recommended [12]. One hundred and fifty minutes of moderate-intensity PA per week is equivalent to 600 Metabolic Equivalent Task (MET)-minutes/week [16].

The opportunity to intervene with PA advice as part of a lung cancer screening program has not been previously explored. The primary objective of this study is to assess the feasibility and safety of recruiting people attending a lung cancer screening trial to participate in a randomised study examining the impact of an 8-week multi-modality exercise program on PA levels and exercise capacity.

## Methods

This feasibility RCT recruited participants from a single Australian International Lung Screen Trial centre (ILST; registered on ClinicalTrials.gov August 2016 NCT02871856). The ILST is a multisite single arm LDCT lung cancer screening study, with the primary objectives of comparing lung cancer screening participant selection criteria and evaluating a nodule management protocol [17]. Participants underwent a baseline LDCT to screen for lung cancer with further interval scans and investigation for lung cancer determined as per the trial protocol using the PanCan nodule calculator score [17]. The ILST recruited women and men aged 55 to 80 years-old who were current or former smokers with either an estimated 6-year lung cancer risk of ≥1.51% (Prostate, Lung, Colorectal and Ovarian 2012 (PLCOm2012) risk prediction model) or ≥30 pack-year smoking history, and Eastern Cooperative Oncology Group (ECOG) performance status 0–1 [17]. Additional RCT inclusion criteria included being able to safely complete home-based exercise, no co-morbidity preventing completion of exercise, no lung cancer diagnosis, and able to walk 100 m independently. Recruitment was planned a 6-month period or until a target of 75 participants were randomised. The study was approved by the Melbourne Health Human Research Ethics Committee (HREC/74684/MH-2021). Written informed consent was obtained from all individuals. This study was registered in July 2022 with Australian Clinical Trials (ACTRN12622001001785). All ILST participants who were currently smoking were offered a referral to a smoking cessation service at the time of enrolment.

### Interventions

Eligible participants were randomised 1:1:1 via computer generated sequence to received either usual care (UC), usual care plus written information (UC + WI), or usual care plus written information and home-based exercise program (UC + WI + EP). The randomisation schedule was generated and uploaded to REDCap by a staff member not involved in the Physical Activity and Lung cancer Screening study.

Usual care consisted of the standard medical and nursing support provided as part of the ILST and included scheduling of LDCTs, communication of LDCT results with primary care providers, and annual written questionnaire follow-up. Written material provided was the Australian physical activity and sedentary behaviour guidelines booklet [18]. This booklet was produced by the Australian Government Department of Health and describes what PA is, its benefits, the recommended levels of PA, suggestions to increase PA, as well as strengthening exercises [18]. The home-based exercise program was individualised to the person (their ability and safety to perform the exercises). The 8-week program was conducted remotely by medical staff trained in the intervention protocol. Medical staff had additional certifications in behavioural change techniques with Health Change Australia [19]. Participants were encouraged to exercise at home unsupervised and received weekly videoconferencing and/or telephone support during the program. The program consisted of educational, resistance training and aerobic components, with weekly progress and goal setting reviews aimed to meet the Australian guideline’s PA targets. The once-a-week session with staff started with a discussion about PA and lung cancer, Australian Guideline recommendations for PA, followed by behavioural change counselling. This included assessment of PA completed during the week, including meeting of any previous PA goals, as well as the participant selecting a new personalised goal for the week. If new resistance exercises were selected as part of the goal, written information, including exercise description, from the Lung Foundation Australia [20] and PhysioTherapy eXercises [21] was emailed to the participant. A full description of the program is summarised in Table E1 (supplementary materials).

### Outcomes

The primary objectives of the study were feasibility and safety of delivering the training program. Feasibility was assessed using consent rate to the study overall, adherence to the exercise program (intervention arm), and attendance at follow-up assessments. A consent rate, exercise adherence rate (measured using home exercise diaries and attendance at weekly review meetings), and attendance at follow-up rate of ≥ 70% was considered a priori to be feasible. This rate of 70% is based on attendance rates reported in prior exercise training studies in the oncology setting [22]. We also collected data on use of the information booklet (read or unread and usefulness) for the relevant two arms at the 9-week timepoint. The participant feedback survey questions are provided in Survey E1 (supplementary materials).

Safety was assessed by the number of adverse events occurring during or within 60 min following the intervention. Information regarding any adverse events was requested from participants during the weekly telephone or telehealth consultation. Serious adverse events are defined as any adverse event related to the study that resulted in death or is life-threatening, requiring hospitalisation, causing disability or incapacity. Minor adverse events were those that are directly caused by exercise such as: a minor fall, new or progressive pain, transient neurological deficits, transient altered mental status, palpitations or progressive fatigue [23].

Secondary objectives were to examine changes in PA, exercise capacity, muscle strength, overall health, and wellbeing measured at three timepoints, baseline, 9 weeks and 6 months and consisted of questionnaires (International Physical Activity Questionnaire (IPAQ) [24], 36-Item Short Form Health Survey (36-SF) [25], Hospital Anxiety and Depression Scale (HADS) [26], and EuroQol five dimensions questionnaire (EQ-5D-5 L) [27] as well as objective assessments (6-minute walk distance (6MWD), hand grip muscle strength, and a 10-day step count measured using an electronic pedometer (Garmin Vivofit 4 or Garmin Vivosmart4). PA levels were measured by IPAQ and step count, whereas 36-SF and EQ-5D-5 L assessed health and wellbeing. HADS specifically assessed mood. Exercise capacity was measured by 6MWD.

### Statistical methods

Data analysis was performed in R version 4.3.1 (R Foundation for Statistical Computing, Vienna, Austria; www.r-project.org). Descriptive statistics were used to summarise demographic characteristics of the participants and the feasibility and safety outcomes. For all numerical outcomes, differences between trial arms at each time point were assessed using one-way ANCOVA with the baseline measurement of each outcome used as a covariate. Residual plots were examined visually to verify the ANCOVA model assumptions of equal variance and approximate normality. Mean differences between each intervention group and the control arm (UC) are reported along with 95% confidence intervals. No p-values are provided as this is a feasibility study with inadequate power to assess the efficacy of the treatment.

## Results

**Fig. 1:**
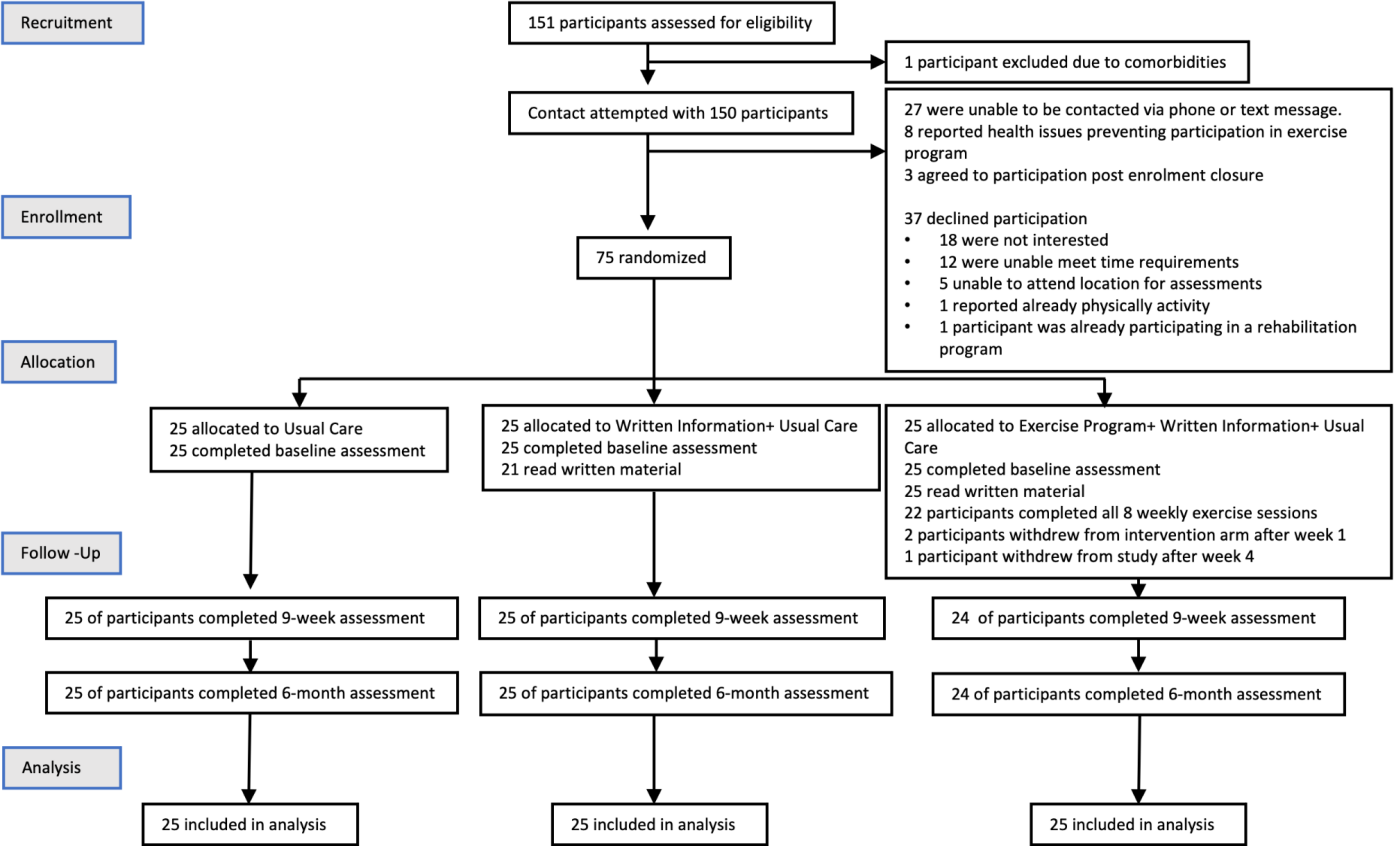
Participant flow diagram

During the recruitment period (May-July 2022), 151 ILST participant records were reviewed. Participant recruitment is described in Fig. 1. 67% of those successfully contacted consented, with an additional three people agreeable to enrol in study after recruitment closure. Of those who declined participation, 49% declined due to disinterest,. 32% declined as they were unable meet intervention time commitment, 14% declined as they were unable to attend onsite for assessments, and a further one participant declined as already physically active.

**Fig. 2:**
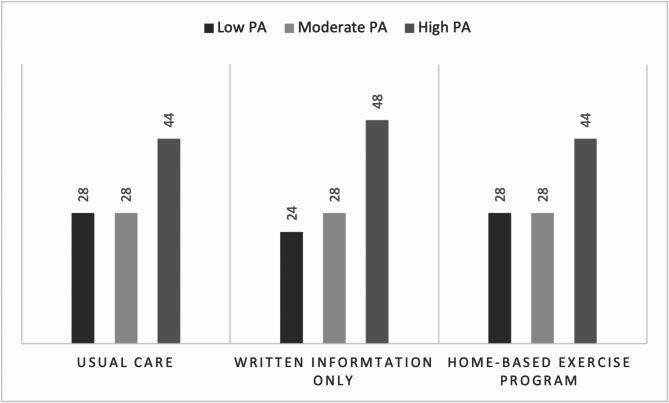
Baseline PA levels by group

Baseline characteristics of those enrolled in the RCT and those who declined are presented in Table E2 (supplementary materials). Characteristics of those enrolled in each study group are presented in Table 1. Baseline PA levels derived from IPAQ for each group are presented in Fig. 2.

**Table 1:** Baseline participant characteristics by group

Characteristic	Usual care group, *N* = 25^*1*^	Written Information only group, *N* = 25^*1*^	Home-based exercise program group, *N* = 25^*1*^
Age	66.0 (63.0, 73.0)	65.0 (62.0, 71.0)	66.0 (61.0, 73.0)
Sex (male)	15 (60%)	13 (52%)	15 (60%)
Education			
8th grade	1 (4%)	0 (0%)	2 (8%)
9th to 11th grade	5 (20%)	7 (28%)	9 (36%)
High school graduate	6 (24%)	6 (24%)	2 (8%)
Technical/vocational certificate	1 (4%)	3 (12%)	2 (8%)
Some college/university	5 (20%)	3 (12%)	2 (8.0%)
University graduate	3 (12%)	2 (8.0%)	5 (20%)
Postgraduate	4 (16%)	4 (16%)	3 (12%)
Work			
Employed	9 (36%)	14 (56%)	7 (28%)
Retired	9 (36%)	7 (32%)	9 (26%)
Disabled	0 (0%)	0 (0%)	2 (8%)
Other	1 (4%)	1 (4%)	1 (4%)
Unemployed	2 (8%)	0 (0%)	1 (4%)
Unknown	4	3	5
Smoking status			
Current	11 (44%)	12 (48%)	11 (44%)
Former	14 (56%)	13 (52%)	14 (56%)
Pack year history	52 (41, 64)	42 (38, 63)	44 (36, 56)
PLCO	3.17 (2.18, 5.34)	2.33 (1.90, 2.87)	2.59 (1.55, 4.20)
FER	72 (62, 78)	68 (65, 71)	69 (65, 77)
FEV1 (%)	103 (84, 109)	99 (90, 103)	95 (83, 109)
DLCO (%)	78 (71, 94)	78 (71, 90)	81 (71, 89)

^*1*^ Median (IQR); n (%)

### Home-based exercise program

Of the 25 participants randomised to the UC + WI + EP arm, 22 participants (88%) attended 100% of their weekly sessions (number attending > 70% of sessions = 22 participants). Twenty-three (92%) elected for their once-a-week review to be via telephone, with the remaining opting for videoconference. Two participants (8%) withdrew from the intervention after attending the first session however continued to have assessment visits, and 1 participant withdrew after week 4 from the study completely. Reasons for withdrawal were collected by staff via telephone at the time of notification and included change in personal commitments and satisfaction with existing PA levels.

69% of weekly PA goals were met by participants. The exercise program was conducted predominantly during Australian winter months. It should be noted that 44% of participants reported being unwell from respiratory viruses including COVID-19 during their program. The most common reason (48%) for not meeting weekly PA goals was illness, followed by busyness (27%), and poor weather (17%). There were no major or minor adverse events associated with the exercise program. Of the 24 participants who completed feedback for the exercise program, 23 (96%) found the program a positive experience, and one participant who withdrew after one session reporting dissatisfaction with use of the pedometer.

### Booklet intervention

100% of UC + WI + EP group and 84% of the UC + WI group reported reading the booklet. 70% (35 participants) reported the booklet was useful.

### Follow-up assessments

99% of participants completed both the 9-week and 6-month follow-up assessments. Key descriptive results over time are presented in Table 2 with the complete EQ-5D-5 L, SF-36 and IPAQ results presented in Table E3 (supplementary materials). Adjusted mean differences between groups (average treatment effects) for secondary outcome measures are summarised in Table E4 (supplementary materials).

**Table 2:** Secondary outcome measures over time by group

	Control			Written information only			Home-based exercise program		
	Baseline^1^	9 weeks^1^	6 months^1^	Baseline^1^	9 weeks^1^	6 months^1^	Baseline^1^	9 weeks^1^	6 months^1^
EQ-5D-5 L	*N* = 25	*N* = 25	*N* = 25	*N* = 25	*N* = 25	*N* = 25	*N* = 25	*N* = 24	*N* = 24
Visual Analogue Scale	77 (16)	75 (19)	74 (18)	82 (12)	81 (11)	79 (13)	77 (17)	74 (19)	77 (17)
HADS	*N* = 25	*N* = 25	*N* = 25	*N* = 25	*N* = 25	*N* = 25	*N* = 25	*N* = 24	*N* = 24
Anxiety	4.9 (3.8)	5.6 (4.5)	5.3 (4.3)	3.7 (3.1)	3.9 (3.8)	3.8 (3.7)	5.44 (3.54)	4.12 (2.61)	4.08 (2.70)
Depression	3.4 (3.1)	4.0 (3.7)	4.0 (3.5)	2.68 (2.27)	2.96 (2.86)	3.00 (2.58)	4.16 (2.25)	2.75 (2.33)	3.29 (2.51)
IPAQ	*N* = 25	*N* = 25	*N* = 25	*N* = 25	*N* = 25	*N* = 25	*N* = 25	*N* = 24	*N* = 24
Total MET/min/week	2,388 (3,150)	1,974 (1,711)	2,156 (1,609)	2,590 (3,129)	1,739 (2,179)	2,224 (2,598)	2,047 (2,240)	3,911 (5,977)	2,611 (2,413)
Sitting Hours	5.5 (3.0)	5.8 (2.9)	5.5 (3.0)	5.2 (2.9)	6.2 (3.5)	5.7 (2.7)	6.8 (4.2)	5.5 (3.5)	5.1 (3.1)
Daily step count	*N* = 25	*N* = 25	*N* = 24	*N* = 25	*N* = 24	*N* = 24	*N* = 25	*N* = 22	*N* = 22
	9,839 (8,536)	9,198 (5,404)	6,997 (3,216)	7,568 (3,919)	8,527 (3,949)	9,097 (4,445)	8,399 (4,286)	9,071 (5,991)	10,029 (5,627)
6MWD	*N* = 24	*N* = 25	*N* = 25	*N* = 25	*N* = 25	*N* = 24	*N* = 25	*N* = 24	*N* = 23
	525 (105)	545 (99)	541 (114)	548 (98)	555 (90)	553 (93)	552 (80)	562 (100)	565 (85)
Hand Grip (males)	*N* = 15	*N* = 15	*N* = 15	*N* = 13	*N* = 13	*N* = 12	*N* = 15	*N* = 15	*N* = 15
Left (kg)	30 (6)	30 (5)	32 (5)	34 (6)	33 (6)	35 (6)	33 (7)	32(7)	32 (6)
Right (kg)	34 (8)	33 (8)	34 (7)	35 (6)	36 (6)	36 (7)	35 (5)	33 (10)	34 (9)
Hand Grip (females)	*N* = 10	*N* = 10	*N* = 10	*N* = 12	*N* = 12	*N* = 12	*N* = 10	*N* = 9	*N* = 8
Left (kg)	15 (5)	15 (5)	15 (5)	18 (5)	19 (5)	20 (5)	18 (4)	21 (6)	21 (5)
Right(kg)	17 (5)	17 (5)	18 (4)	22 (5)	23 (5)	22 (6)	20 (6)	23(7)	24 (6)

^1^ Mean (SD)

**Fig. 3:**
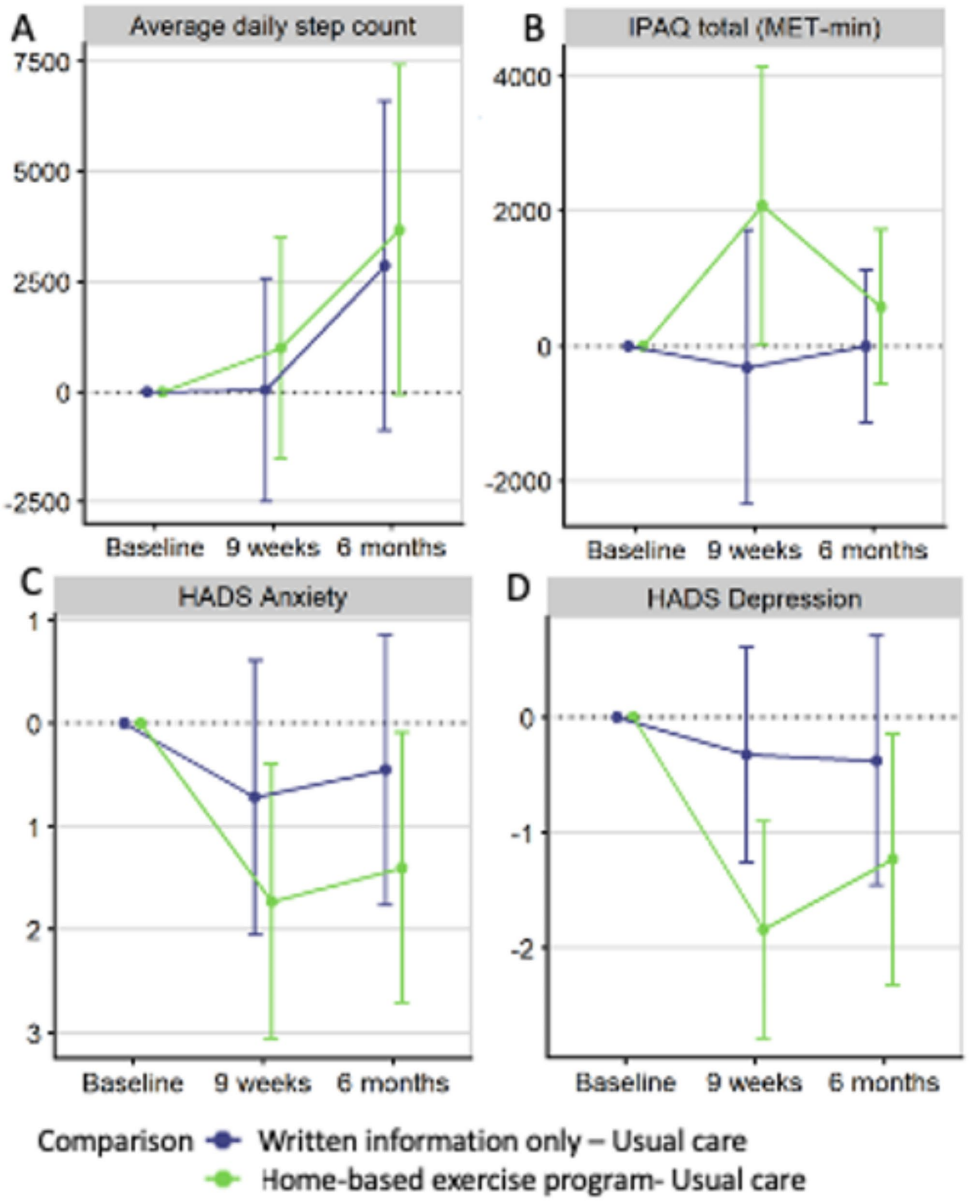
Adjusted mean differences between groups with 95% confidence intervals for average daily step count (**A**), total IPAQ score (**B**), HADS Anxiety score (**C**), and HADS Depression score (**D**). Each variable is adjusted for baseline values of that variable using ANCOVA

As this was a feasibility study, it was not powered to detect meaningful differences in secondary outcomes. Our cohort had a similar baseline overall health and wellbeing scores (EQ-5D-5 L VAS and SF-36) compared to the general Australian population [28, 29]. While the uncertainty in these outcomes was substantial, the results were consistent with improved physical activity levels (step count and IPAQ scores) as depicted in Fig. 3A and B in the UC + WI + EP group compared with the UC group at 9-week and 6-months. Reduction in HADS anxiety and depression scores were also noted (Fig. 3C and D), however these were not clinically significant. There were no obvious patterns noted in the UC + WI group compared to UC group.

As an exploratory investigation, progress over time for the UC + WI + EP group by baseline IPAQ PA intensity is presented in Figure E1 (supplementary materials) for total IPAQ score, 6MWD, daily step count and grip strength. While no statistical comparisons were performed, it was observed that participants of the UC + WI + EP with low PA levels at baseline also had improved PA levels (total IPAQ) and step counts which while lower than at 9 weeks, were still higher than baseline at 6 months.

## Discussion

To our knowledge, this phase 1 feasibility RCT is the first to evaluate any kind of exercise intervention aimed at improving PA levels in high-risk individuals with active smoking history as part of a lung cancer screening program. This study demonstrated that screening participants were interested in a PA intervention when offered as part of lung cancer screening. The overall consent rate of those contacted for the study was 67%. Whilst this is marginally lower than the pre-specified 70%, recruitment to the study was closed after only 3 months due to meeting pre-defined capacity. There was high compliance with the home-based exercise program (88% of participants attending 100% of weekly sessions), and attendance of follow-up (99% participants completed all three assessments). The program was safe, with no adverse events reported.

There is a paucity of published data prospectively evaluating PA levels in people at high-risk of lung cancer. A Canadian case-control study with 2078 participants reported a lower lung cancer risk in both men (OR 0.66, 95% CI 0.46–0.92) and women (OR 0.55, 95% CI 0.34–0.88) in the highest tertile of recreational PA compared with those in the lowest [30]. We have previously conducted a prospective observational study of PA levels in our lung cancer screening cohort which demonstrated that 24% of our cohort did not meet WHO and Australian guideline recommendations for PA [31]. Whist screening trials are at risk of healthy volunteer bias, there is still a significant proportion of high-risk individuals who have low PA levels which is a potentially modifiable risk factor for lung cancer. Additionally there are also the well-established benefits of PA for cancer-mortality, all-cause mortality, and cardiovascular health in older adults [32].

Similar exercise programs have been trialled in the lung cancer setting [33]. As highlighted by the CAPACITY study, sustained behaviour changes and improvements in PA are difficult to maintain [33]. However, the incorporation of behavioural change strategies including overcoming barriers in a self-directed manner and self-designed structured weekly goals is a strength of this intervention [19, 34].

### Limitations

This was a feasibility study aimed at evaluating recruitment, adherence, and intervention safety, and therefore enrolment contact was attempted consecutively and/or at existing follow-up appointments. As such, the majority of our study cohort met PA guidelines at baseline. In these instances, participants were offered to increase the duration and/or intensity of PA, focus on endurance and strengthening, or maintain current PA goals. In future studies aimed at evaluating efficacy, incorporation of baseline PA levels to determine eligibility would be desired to ensure the intervention is improving baseline low levels of PA [35]. This study did not collect information regarding smoking cessation attempts; however, this would be useful in future research given previously demonstrated benefits of PA on smoking patterns. Due to the nature of the intervention, study participants and staff were not blinded to the intervention. A high proportion of participants also experienced respiratory illness during the exercise program impacting progress. This is likely related to the winter season.

In conclusion, this study demonstrated that a home-based exercise program is feasible and safe and could be a low-risk, intervention with significant benefits for high-risk individuals participating in a lung cancer screening program. Like with smoking cessation advice, the opportunity to intervene with PA advice at the point of lung cancer risk assessment of people with an active smoking history, even in those who may not be eligible for screening, may be invaluable. Further evaluation of the home-based exercise program is needed to determine efficacy as part of a lung cancer screening program.

## Electronic supplementary material

Below is the link to the electronic supplementary material.

Supplementary Material 1

## Data Availability

The datasets analysed during the current study are available from the corresponding author on reasonable request.

## References

[1] Sung H, Ferlay J, Siegel RL, Laversanne M, Soerjomataram I, Jemal A, et al. Global Cancer statistics 2020: GLOBOCAN estimates of incidence and mortality worldwide for 36 cancers in 185 countries. CA Cancer J Clin. 2021;71(3):209–49. 33538338 10.3322/caac.21660

[2] Batty GD, Russ TC, Stamatakis E, Kivimäki M. Psychological distress in relation to site specific cancer mortality: pooling of unpublished data from 16 prospective cohort studies. BMJ. 2017;356:j108. 28122812 10.1136/bmj.j108 PMC5266623

[3] Granger C, McDonald C, Irving L, Clark R, Gough K, Murnane A, et al. Low physical activity levels and functional decline in individuals with lung cancer. Lung cancer (Amsterdam Netherlands). 2014;83(2):292–9. 24360323 10.1016/j.lungcan.2013.11.014

[4] Bonney A, Malouf R, Marchal C, Manners D, Fong KM, Marshall HM, et al. Impact of low-dose computed tomography (LDCT) screening on lung cancer-related mortality. Cochrane Database Syst Rev. 2022;8(8):Cd013829. 35921047 10.1002/14651858.CD013829.pub2 PMC9347663

[5] Network LCP. Interactive map of lung cancer screening: Lung Cancer Policy Network; 2024 [updated December 2024. Second:[Available from: https://www.lungcancerpolicynetwork.com/interactive-map-of-lung-cancer-screening/

[6] Cadham CJ, Jayasekera JC, Advani SM, Fallon SJ, Stephens JL, Braithwaite D, et al. Smoking cessation interventions for potential use in the lung cancer screening setting: A systematic review and meta-analysis. Lung cancer (Amsterdam Netherlands). 2019;135:205–16. 31446996 10.1016/j.lungcan.2019.06.024 PMC6739236

[7] Liu Y, Li Y, Bai YP, Fan XX. Association between physical activity and lower risk of lung cancer: A Meta-Analysis of cohort studies. Front Oncol. 2019;9:5. 30723700 10.3389/fonc.2019.00005 PMC6349707

[8] Buffart LM, Singh AS, van Loon EC, Vermeulen HI, Brug J, Chinapaw MJ. Physical activity and the risk of developing lung cancer among smokers: a meta-analysis. J Sci Med Sport. 2014;17(1):67–71. 23528254 10.1016/j.jsams.2013.02.015

[9] Schmid D, Ricci C, Behrens G, Leitzmann MF. Does smoking influence the physical activity and lung cancer relation? A systematic review and meta-analysis. Eur J Epidemiol. 2016;31(12):1173–90. 27502335 10.1007/s10654-016-0186-y

[10] Sun S, Schiller JH, Gazdar AF. Lung cancer in never smokers–a different disease. Nat Rev Cancer. 2007;7(10):778–90. 17882278 10.1038/nrc2190

[11] Filaire E, Dupuis C, Galvaing G, Aubreton S, Laurent H, Richard R, et al. Lung cancer: what are the links with oxidative stress, physical activity and nutrition. Lung cancer (Amsterdam Netherlands). 2013;82(3):383–9. 24161719 10.1016/j.lungcan.2013.09.009

[12] Bull FC, Al-Ansari SS, Biddle S, Borodulin K, Buman MP, Cardon G, et al. World health organization 2020 guidelines on physical activity and sedentary behaviour. Br J Sports Med. 2020;54(24):1451–62. 33239350 10.1136/bjsports-2020-102955 PMC7719906

[13] Zhou Y, Feng W, Guo Y, Wu J. Effect of exercise intervention on smoking cessation: a meta-analysis. Front Physiol. 2023;14:1221898. 37614760 10.3389/fphys.2023.1221898 PMC10442508

[14] Reduced Lung-Cancer Mortality with Low-Dose Computed Tomographic Screening. N Engl J Med. 2011;365(5):395–409. 21714641 10.1056/NEJMoa1102873 PMC4356534

[15] Granger CL, McDonald CF, Berney S, Chao C, Denehy L. Exercise intervention to improve exercise capacity and health related quality of life for patients with Non-small cell lung cancer: a systematic review. Lung cancer (Amsterdam Netherlands). 2011;72(2):139–53. 21316790 10.1016/j.lungcan.2011.01.006

[16] Armstrong T, Bull F. Development of the world health organization global physical activity questionnaire (GPAQ). J Public Health. 2006;14:66–70.

[17] Lim KP, Marshall H, Tammemägi M, Brims F, McWilliams A, Stone E, et al. Protocol and rationale for the international lung screening trial. Ann Am Thorac Soc. 2020;17(4):503–12. 32011914 10.1513/AnnalsATS.201902-102OC PMC7175983

[18] Department of Health. In: Government A, editor. Australia’s physical activity and sedentary behaviour guidelines. Australia: Commonwealth of Australia; 2014.

[19] Gale J, Skouteris H. Health coaching: facilitating health behavior change for chronic condition prevention and self-management. Applied topics in health psychology. Wiley-Blackwell; 2013. pp. 15–28.

[20] Lung Foundation Australia. Better Living with Exercise: Lung Foundation Australia; [Available from: https://lungfoundation.com.au/resources/better-living-with-exercise-booklet/

[21] NSW Department of Health. PhysioTherapy eXercises for people with injuries and disabilities Australia: NSW Department of Health [Available from: https://www.physiotherapyexercises.com/

[22] Lu TDL, Cao Y, Cong Q, Wu E, Granger CL, Ni J, Edbrooke L. A 12-Week Multi-Modal exercise program: feasibility of combined exercise and simplified 8-Style Tai Chi following lung Cancer surgery. Integr Cancer Ther. 2020;19:1–11. 10.1177/1534735420952887 PMC7457649 32851871

[23] Edbrooke LAS, Granger CL, McDonald CF, Krishnasamy M, Mileshkin L, Irving L, Braat S, Clark RA, Gordon I, Denehy L. Benefits of home-based multidisciplinary exercise and supportive care in inoperable non-small cell lung cancer - protocol for a phase II randomised controlled trial. BMC Cancer. 2017;17(1). 10.1186/s12885-017-3651-4 PMC5622453 28962608

[24] Craig CL, Marshall AL, Sjostrom M, Bauman AE, Booth ML, Ainsworth BE, et al. International physical activity questionnaire: 12-country reliability and validity. Med Sci Sports Exerc. 2003;35(8):1381–95. 12900694 10.1249/01.MSS.0000078924.61453.FB

[25] Ware JE. SF-36 health survey. Manual and interpretation guide. Health Inst. 1993;6(1–6):22.

[26] Bjelland I, Dahl AA, Haug TT, Neckelmann D. The validity of the hospital anxiety and depression scale: an updated literature review. J Psychosom Res. 2002;52(2):69–77. 11832252 10.1016/s0022-3999(01)00296-3

[27] Herdman M, Gudex C, Lloyd A, Janssen MF, Kind P, Parkin D, et al. Development and preliminary testing of the new five-level version of EQ-5D (EQ-5D-5L). Qual Life Res. 2011;20(10):1727–36. 21479777 10.1007/s11136-011-9903-x PMC3220807

[28] McCaffrey N, Kaambwa B, Currow DC, Ratcliffe J. Health-related quality of life measured using the EQ-5D–5L: South Australian population norms. Health Qual Life Outcomes. 2016;14(1):133. 27644755 10.1186/s12955-016-0537-0 PMC5028927

[29] Stevenson C. SF-36: Interim norms for Australian data. Canberra, Australia. 1996.

[30] Ho V, Parent ME, Pintos J, Abrahamowicz M, Danieli C, Richardson L, et al. Physical activity and lung cancer risk in men and women. Cancer Causes Control. 2017;28(4):309–18. 28247218 10.1007/s10552-017-0872-4

[31] Bonney AGC, Steinfort D, Marshall HM, Stone E, McWilliams A, Brims F, Fogarty P, Lin L, Li J, Pang S, Lam S, Fong KM, Manser R. Brief report: A prospective observational study of physical activity levels and physical fitness of people at high-risk for lung cancer. J Thorac Oncol Clin Res Rep 2024;Article in press. 10.1016/j.jtocrr.2024.100633 PMC10874747 38371193

[32] Watts EL, Matthews CE, Freeman JR, Gorzelitz JS, Hong HG, Liao LM, et al. Association of leisure time physical activity types and risks of All-Cause, cardiovascular, and Cancer mortality among older adults. JAMA Netw Open. 2022;5(8):e2228510. 36001316 10.1001/jamanetworkopen.2022.28510 PMC9403775

[33] Granger CL, Irving L, Antippa P, Edbrooke L, Parry SM, Krishnasamy M, et al. CAPACITY: A physical activity self-management program for patients undergoing surgery for lung cancer, a phase I feasibility study. Lung cancer (Amsterdam Netherlands). 2018;124:102–9. 30268446 10.1016/j.lungcan.2018.07.034

[34] Troosters T, Blondeel A, Rodrigues FM, Janssens W, Demeyer H. Strategies to increase physical activity in chronic respiratory diseases. Clin Chest Med. 2019;40(2):397–404. 31078217 10.1016/j.ccm.2019.02.017

[35] Avancini A, Belluomini L, Quist M, Pilotto S. Lung Cancer screening: an opportunity to promote physical activity?? JTO Clin Res Rep. 2024;5(3):100651. 38496375 10.1016/j.jtocrr.2024.100651 PMC10940996

