# Supplementary material for: Physical activity and lung cancer screening (PALS): feasibility randomised controlled trial of exercise and physical activity in lung cancer screening
Source: Respir Res. 2025 Mar 6;26:89. doi: 10.1186/s12931-025-03158-0 (PMC11884085; doi:10.1186/s12931-025-03158-0)
Supplement: Supplementary file 1 — Supplementary Material 1 [file 12931_2025_3158_MOESM1_ESM.docx]

**Physical Activity and Lung cancer Screening (PALS): a feasibility randomised controlled trial of exercise and physical activity in lung cancer screening.**

**Online Data Supplement.**

**Table E1. Components of the 8-week home-based exercise program**

| Component | Frequency | Duration | Intensity | Type | Progression |
| --- | --- | --- | --- | --- | --- |
| *Education* | | | | | |
| Discussion of role of PA in people at risk of lung cancer | Once a week | <15 minutes | N/A | N/A | N/A |
| Review PA guideline recommendations | Once a week | <15 minutes | N/A | N/A | N/A |
| Written information booklet | Program start | <15 minutes | N/A | N/A | N/A |
| *Interventions* | | | | | |
| Aerobic exercise | 5 days/ week | Progress to ≥30 minutes | At least moderate intensity continuous exercise rated with Borg dyspnoea scale (4 ‘somewhat hard’)(E1) | Walking, running, cycling, water aerobics, swimming | Increase time and speed to maintain intensity of exercise |
| Resistance exercise | 2-3 days/ week | Each exercise: 10 reps, 2-3 sets | Moderate intensity continuous exercise rated with Borg dyspnoea scale (4 ‘somewhat hard’)(E1) | Lower limb functional exercises using body weight: squats, step-ups, sit-to-stand, heel raises, leg extension.  Upper limb exercises: shoulder press, bicep curl, side lateral raise, wall push-up  Load that can be achieved in 10 reps maximum | Increase reps to 10 per exercise then increase sets to 3 per exercise, then increase weight. |
| Behavioural change counselling | Once a week | 5-15 minutes | N/A | Consultation focused on motivation for change, goal setting, barriers/enablers to exercise, and confidence in achieving goals | N/A |

**Table E2. Characteristics by enrolment status.**

| ****Characteristic**** | ****Not enrolled****, N = 76*^1^* | ****Enrolled****, N = 75*^1^* |
| --- | --- | --- |
| Age | 65.0 (61.0, 71.0) | 66.0 (62.0, 72.5) |
| Sex (male) | 44 (58%) | 43 (57%) |
| Education |  |  |
| 8th grade | 2 (3%) | 3 (4%) |
| 9th to 11th grade | 25 (33%) | 21 (28%) |
| High school graduate | 14 (18%) | 14 (19%) |
| Technical/vocational certificate | 6 (8%) | 6 (8%) |
| Some college/university | 9 (12%) | 10 (13%) |
| University graduate | 10 (13%) | 10 (13%) |
| Postgraduate | 10 (13%) | 11 (15%) |
| Work |  |  |
| Working | 27 (45%) | 30 (48%) |
| Retired | 22 (37%) | 25 (40%) |
| Disabled | 4 (7%) | 2 (3%) |
| Other | 3 (5%) | 3 (5%) |
| Unemployed | 4 (7%) | 3 (5%) |
| Unknown | 16 | 12 |
| Smoking status |  |  |
| Current | 43 (57%) | 34 (45%) |
| Former | 33 (43%) | 41 (55%) |
| Pack year history | 42 (32, 54) | 46 (38, 61) |
| PLCO | 3.03 (1.59, 4.33) | 2.59 (1.93, 4.78) |
| FER | 71 (67, 76) | 69 (65, 76) |
| FEV1 (%) | 99 (87, 110) | 97 (86, 108) |
| DLCO (%) | 77 (68, 87) | 79 (71, 91) |

1 Median (IQR); n (%)

**Table E3. Completed EQ-5D-5L, SF-36 and IPAQ measures over time by group.**

|  | Control | | | Written information only | | | Home-based exercise program | | |
| --- | --- | --- | --- | --- | --- | --- | --- | --- | --- |
|  | Baseline^1^ | 9 weeks^1^ | 6 months^1^ | Baseline^1^ | 9 weeks^1^ | 6 months^1^ | Baseline^1^ | 9 weeks^1^ | 6 months^1^ |
| EQ-5D-5L | N=25 | N=25 | N=25 | N=25 | N=25 | N=25 | N=25 | N=24 | N=24 |
| Mobility | 1.68 (0.85) | 1.68 (0.75) | 1.72 (0.84) | 1.60 (0.76) | 1.44 (0.77) | 1.60 (0.87) | 1.40 (0.58) | 1.42 (0.58) | 1.71 (0.91) |
| Personal Care | 1.32 (0.63) | 1.16 (0.37) | 1.16 (0.47) | 1.08 (0.28) | 1.04 (0.20) | 1.08 (0.28) | 1.08 (0.28) | 1.13 (0.34) | 1.25 (0.44) |
| Usual Activity | 1.68 (0.90) | 1.60 (0.82) | 1.60 (0.71) | 1.40 (0.65) | 1.32 (0.56) | 1.48 (0.65) | 1.48 (0.65) | 1.29 (0.46) | 1.54 (0.66) |
| Pain/ Discomfort | 2.20 (0.76) | 2.24 (0.88) | 2.20 (0.71) | 2.04 (0.54) | 1.92 (0.64) | 1.80 (0.58) | 2.24 (0.60) | 1.96 (0.75) | 2.33 (0.82) |
| Anxiety/ Depression | 1.64 (0.70) | 1.84 (0.94) | 1.60 (0.76) | 1.44 (0.65) | 1.36 (0.70) | 1.48 (0.65) | 1.64 (0.70) | 1.46 (0.72) | 1.54 (0.72) |
| Visual Analogue Scale | 77 (16) | 75 (19) | 74 (18) | 82 (12) | 81 (11) | 79 (13) | 77 (17) | 74 (19) | 77 (17) |
| SF-36 | N=25 | N=25 | N=25 | N=25 | N=25 | N=25 | N=25 | N=24 | N=24 |
| Physical Functioning | 71 (25) | 74 (23) | 72 (24) | 71 (25) | 75 (20) | 76 (21) | 78 (18) | 80 (15) | 76 (20) |
| Physical Health Limitations | 71 (39) | 69 (39) | 63 (38) | 76 (36) | 68 (41) | 65 (40) | 80 (32) | 61 (41) | 67 (40) |
| Emotional Limitations | 67 (41) | 77 (38) | 80 (32) | 87 (27) | 88 (27) | 77 (34) | 77 (37) | 94 (16) | 74 (37) |
| Energy/ Fatigue | 54 (19) | 54 (23) | 53 (22) | 62 (18) | 60 (20) | 59 (19) | 58 (16) | 61 (16) | 58 (20) |
| Emotional Wellbeing | 74 (17) | 74 (18) | 76 (17) | 82 (11) | 81 (17) | 78 (18) | 77 (15) | 81 (12) | 75 (16) |
| Social Functioning | 80 (24) | 79 (26) | 81 (25) | 92 (12) | 85 (22) | 82 (21) | 81 (22) | 89 (17) | 85 (20) |
| Pain | 67 (26) | 74 (23) | 71 (26) | 76 (16) | 76 (19) | 81 (19) | 68 (20) | 69 (17) | 63 (25) |
| General Health | 57 (21) | 60 (21) | 56 (23) | 66 (19) | 63 (14) | 64 (19) | 62 (14) | 66 (14) | 64 (15) |
| IPAQ | N=25 | N=25 | N=25 | N=25 | N=25 | N=25 | N=25 | N=24 | N=24 |
| Vigorous MET/min/week | 131 (257) | 112 (169) | 88 (126) | 104 (205) | 75 (171) | 83 (183) | 60 (141) | 216 (425) | 123 (165) |
| Moderate MET/min/week | 106 (199) | 93 (117) | 149 (157) | 195 (300) | 107 (161) | 172 (239) | 162 (283) | 262 (643) | 136 (262) |
| Light MET/min/week | 278 (251) | 214 (154) | 259 (291) | 298 (503) | 215 (197) | 264 (228) | 278 (340) | 344 (287) | 328 (298) |
| Total MET/min/week | 2,388 (3,150) | 1,974 (1,711) | 2,156 (1,609) | 2,590 (3,129) | 1,739 (2,179) | 2,224 (2,598) | 2,047 (2,240) | 3,911 (5,977) | 2,611 (2,413) |
| Sitting Hours | 5.5 (3.0) | 5.8 (2.9) | 5.5 (3.0) | 5.2 (2.9) | 6.2 (3.5) | 5.7 (2.7) | 6.8 (4.2) | 5.5 (3.5) | 5.1 (3.1) |

^1^ Mean (SD)

N=number of participants included

**Table E4. Summary of secondary outcome measures.**

|  | Time | Written information only^1^ | Home-based exercise program^1^ |
| --- | --- | --- | --- |
| EQ-5D-5L |  |  |  |
| Mobility | 9 weeks | -0.19 (-0.48, 0.10) | -0.09 (-0.39, 0.21) |
|  | 6 months | -0.05 (-0.40, 0.30) | 0.21 (-0.15, 0.56) |
| Personal Care | 9 weeks | -0.01 (-0.16, 0.13) | 0.09 (-0.06, 0.24) |
|  | 6 months | 0.04 (-0.16, 0.25) | 0.23 (0.03, 0.44) |
| Usual Activity | 9 weeks | -0.13 (-0.42, 0.16) | -0.17 (-0.46, 0.12) |
|  | 6 months | 0.02 (-0.31, 0.35) | 0.07 (-0.26, 0.40) |
| Pain/ Discomfort | 9 weeks | -0.20 (-0.53, 0.14) | -0.29 (-0.63, 0.05) |
|  | 6 months | -0.31 (-0.67, 0.04) | 0.13 (-0.23, 0.48) |
| Anxiety/ Depression | 9 weeks | -0.32 (-0.65, 0.01) | -0.37 (-0.70, -0.04) |
|  | 6 months | 0.02 (-0.27, 0.32) | -0.05 (-0.34, 0.25) |
| Visual Analogue Scale | 9 weeks | 3.63 (-4.92, 12.17) | -0.96 (-9.53, 7.61) |
|  | 6 months | 1.36 (-4.45, 7.17) | 2.90 (-2.93, 8.73) |
| HADS |  |  |  |
| Anxiety | 9 weeks | -0.72 (-2.05, 0.61) | -1.73 (-3.06, -0.40) |
|  | 6 months | -0.45 (-1.76, 0.86) | -1.40 (-2.71, -0.09) |
| Depression | 9 weeks | -0.45 (-1.76, 0.86) | -1.40 (-2.71, -0.09) |
|  | 6 months | -0.38 (-1.46, 0.70) | -1.24 (-2.32, -0.15) |
| SF-36 |  |  |  |
| Physical Functioning | 9 weeks | 0.65 (-7.33, 8.63) | 1.43 (-6.70, 9.57) |
|  | 6 months | 3.83 (-5.72, 13.38) | -0.20 (-9.93, 9.54) |
| Physical Health Limitations | 9 weeks | -3.68 (-24.09, 16.74) | -14.14 (-34.95, 6.67) |
|  | 6 months | -0.40 (-20.79, 19.99) | -2.26 (-23.04, 18.52) |
| Emotional Limitations | 9 weeks | 2.13 (-12.05, 16.30) | 12.37 (-1.69, 26.42) |
|  | 6 months | -10.91 (-29.03, 7.20) | -10.97 (-28.94, 7.00) |
| Energy/ Fatigue | 9 weeks | -0.19 (-8.61, 8.22) | 5.11 (-3.26, 13.49) |
|  | 6 months | -0.89 (-9.04, 7.25) | 2.26 (-5.83, 10.36) |
| Emotional Wellbeing | 9 weeks | 0.22 (-4.85, 5.28) | 4.18 (-0.82, 9.19) |
|  | 6 months | -5.67 (-11.68, 0.34) | -4.92 (-10.85, 1.02) |
| Social Functioning | 9 weeks | -1.65 (-12.31, 9.01) | 7.76 (-2.69, 18.22) |
|  | 6 months | -5.32 (-16.66, 6.03) | 2.43 (-8.69, 13.54) |
| Pain | 9 weeks | -2.41 (-12.53, 7.71) | -6.22 (-16.28, 3.84) |
|  | 6 months | 4.91 (-6.41, 16.23) | -9.55 (-20.80, 1.71) |
| General Health | 9 weeks | -3.07 (-10.00, 3.86) | 2.69 (-4.21, 9.60) |
|  | 6 months | 0.59 (-6.54, 7.73) | 3.97 (-3.13, 11.08) |
| IPAQ |  |  |  |
| Vigorous (MET/min/week) | 9 weeks | -25.04 (-175.62, 125.55) | 133.08 (-20.23, 286.40) |
|  | 6 months | 5.61 (-71.66, 82.89) | 62.29 (-16.39, 140.97) |
| Moderate (MET/min/week) | 9 weeks | 6.90 (-212.96, 226.76) | 166.03 (-54.55, 386.62) |
|  | 6 months | 10.21 (-115.92, 136.33) | -19.65 (-146.19, 106.89) |
| Light (MET/min/week) | 9 weeks | -6.10 (-101.59, 89.39) | 129.40 (32.94, 225.86) |
|  | 6 months | -2.60 (-131.50, 126.31) | 68.39 (-61.82, 198.61) |
| Total (MET/min/week) | 9 weeks | -317.02 (-2350.30, 1716.26) | 2078.19 (22.15, 4134.22) |
|  | 6 months | -5.76 (-1134.84, 1123.32) | 581.27 (-560.45, 1722.99) |
| Sitting Hours | 9 weeks | 0.54 (-1.07, 2.15) | -0.91 (-2.58, 0.75) |
|  | 6 months | 0.36 (-0.82, 1.55) | -1.22 (-2.44, 0.01 |
| Daily step count | 9 weeks | 38.65 (-2500.43, 2577.73) | 994.43 (-1514.46, 3503.32) |
|  | 6 months | 2854.18 (-881.08, 6589.45) | 3670.92 (-67.71, 7409.56) |
| 6MWD | 9 weeks | -9.50 (-42.45, 23.44) | -4.26 (-37.57, 29.04) |
|  | 6 months | -3.50 (-36.44, 29.45) | -1.33 (-34.80, 32.14) |
| Hand Grip |  |  |  |
| Left (kg) | 9 weeks | 0.46 (-1.78, 2.71) | 1.76 (-0.52, 4.03) |
|  | 6 months | 0.89 (-1.65, 3.43) | -0.44 (-3.03, 2.16) |
| Right (kg) | 9 weeks | 1.50 (-1.80, 4.80) | 0.39 (-2.95, 3.74) |
|  | 6 months | 0.42 (-2.56, 3.40) | 0.11 (-2.93,3.15) |

^1^ Adjusted mean difference between usual care group (95% confidence interval)

**Survey E1. Participant Feedback**

1. Which group were you in?
2. Do you have any feedback for the overall trial?
3. If assigned to a group with written information.
   1. Did you read the booklet?
   2. Did you find the booklet useful?
   3. Do you have any feedback for the written information?
4. If assigned to the exercise program.
   1. Did you did you find the exercise program useful?
   2. Did you have any feedback for the exercise program?

**Figure E1. Individual exercise group participant changes over time for total IPAQ score, average daily step count, grip strength (left hand), and 6MWD by IPAQ PA intensity. Each line represents an individual participant’s outcome. Line colours are arbitrary and intended to help distinguish participants only.**

**
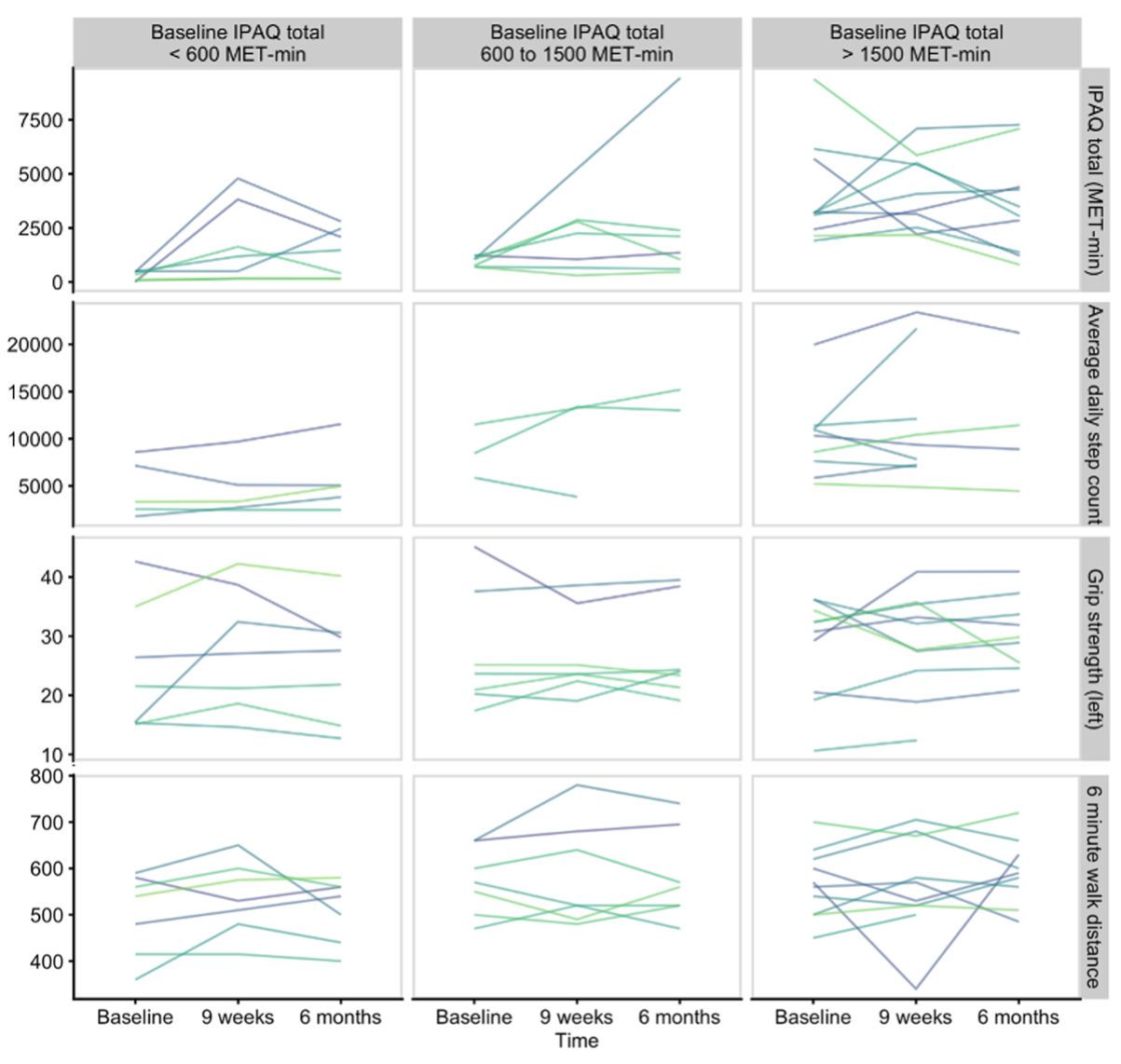
**

**References**

E1. Borg G. Ratings of perceived exertion and heart rates during short-term cycle exercise and their use in a new cycling strength test. *Int J Sports Med* 1982; 3: 153-158.
